# Surgical resection of spinal dural arteriovenous fistula with micro-tubular retractors under CT guidance: cases report and literature review

**DOI:** 10.3389/fsurg.2026.1857509

**Published:** 2026-07-24

**Authors:** Wei Liu, Shengzi Dongye, Yuanqin Liu, Yanda Lin, Feng Li

**Affiliations:** 1 Department of Neurosurgery, The First Affiliated Hospital of Shandong First Medical University and Shandong Provincial Qianfoshan Hospital, Jinan, China; 2 Department of Neurology, The Second Affiliated Hospital of Shandong First Medical University, Taian, Shandong, China

**Keywords:** CT guidance, indocyanine green angiography, micro-tubular retractors, minimally invasive surgery, spinal dural arteriovenous fistula (SDAVF)

## Abstract

**Objective:**

This case report describes the surgical management of spinal dural arteriovenous fistulas (SDAVFs) using micro-tubular retractors under CT guidance, along with a review and analysis of the relevant literature.

**Methods:**

The surgical approach involved the use of micro-tubular retractors for a minimally invasive microsurgical technique, guided by CT imaging to treat two cases of spinal dural arteriovenous fistula (SDAVF). The procedure included intraoperative computed tomography (CT) for precise localization of the fistula and indocyanine green angiography to confirm the position and obliteration of the fistula. Preoperative symptoms and postoperative outcomes were documented.

**Results:**

Complete obliteration of the fistula was achieved in both patients through minimally invasive microsurgery via a micro-tubular retractor. Clinical improvement was observed postoperatively, and no complications related to the surgical procedure were noted. Early postoperative follow-up (during hospitalization) demonstrated no recurrence of the fistula or postoperative complications.Traditional open surgery may compromise spinal stability; however, the use of a micro-tubular retractor significantly mitigates this risk.

**Conclusion:**

Microsurgery via a micro-tubular retractor under CT guidance is a feasible and effective approach for the surgical treatment of spinal dural arteriovenous fistulas (SDAVFs). This method achieves precise localization and minimizes surgical trauma by reducing damage to muscles, ligaments, and bony structures, thereby shortening postoperative recovery time. However, long-term follow-up and additional cases are needed to further validate the clinical efficacy of this technique.

## Introduction

Spinal dural arteriovenous fistula (SDAVF) is the most common spinal vascular malformation account for approximately 70%–80% (1), it is still rare, and the etiology of which is not entirely clear. Epidemiological investigations have shown that the incidence of SDAVF is 5–10 per million per year (2). The major clinical manifestations of SDAVF include motor, sensory, and sexual dysfunction with significant neurological consequences if left untreated (3, 4). Misdiagnosis as a spinal degenerative disorder is common due to its non-specific clinical features (5), a study conducted in 2019 found that 78% SDAVFs were misdiagnosed, and leading to irreversible neurological dysfunction (6). Treatment options include endovascular embolization and operation include microsurgery, and the choice remains somewhat controversial. In the present two cases, surgical treatment was preferred over endovascular embolization for the following reasons: (1) the fistulas were single-level lesions readily accessible via a minimally invasive posterior approach, (2) the angioarchitecture on preoperative DSA did not demonstrate anatomical features that would preclude safe surgical obliteration, and (3) surgical ligation offers the advantages of direct visualization, immediate confirmation of fistula obliteration via intraoperative ICG angiography, and a well-documented favorable long-term occlusion rate (7). We recently performed SDAVF surgery in two patients using a modified microtubular approach and report our experience here.

A literature review was conducted to identify published cases of SDAVF treated with minimally invasive surgical approaches. The PubMed, Embase, and Web of Science databases were searched from inception to May 2025 using the following keywords: “spinal dural arteriovenous fistula,” “SDAVF,” “surgery,” “microsurgery,” “minimally invasive,” “tubular retractor,” “micro-tubular,” and “microchannel.” Studies were included if they described minimally invasive surgical techniques for the treatment of SDAVF. After removing duplicates and screening, three studies reporting minimally invasive surgical approaches for SDAVF were identified (Table 1), together with the two cases presented in this report.

**Table 1:** Summary of reported cases of SDAVF treated with minimally invasive surgery.

Author/Year	Cases	Diagnosis	Treatment	Age/Sex	Outcome
Present Case 1	1	SDAVF, thoracolumbar	CT-guided tubular retractor microsurgery + ICG	44/M	Symptom improvement at 2 d post-op; no complications
Present Case 2	1	SDAVF, thoracic	CT-guided tubular retractor microsurgery + ICG	73/M	Symptom improvement at 2 d post-op; no complications
Msheik et al., 2023 (3)	1	T11–T12 SDAVF	Open microsurgical disconnection	43/M	Improvement in lower limb weakness and numbness
Wang et al., 2023 (8)	1	Intracranial DAVF + SDAVF	Open surgery (lumbar SDAVF)	57/M	Partial improvement after SDAVF surgery

### Case 1

**Figure 1:**
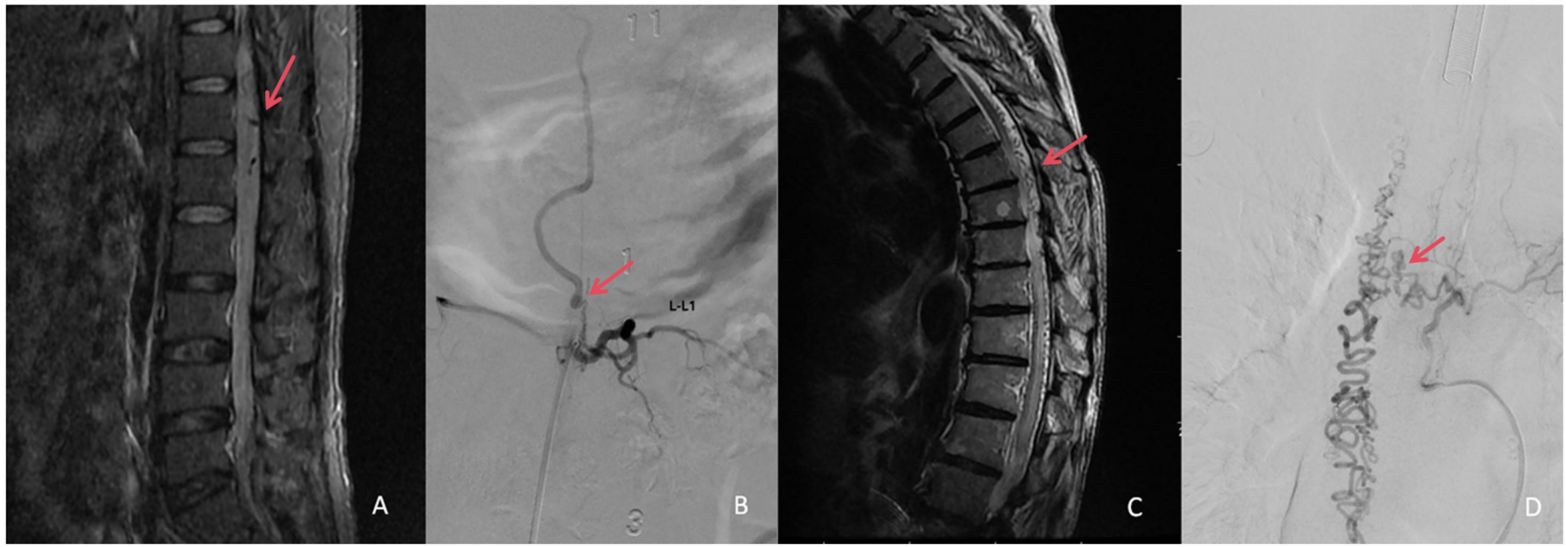
The images depict the MRI and angiographic findings of two patients. **(A)** The sagittal T2-weighted MRI of the first case reveals prominent perimedullary flow voids in the thoracolumbar region. **(B)** The results of digital subtraction angiography (DSA) for the first case, with the fistulous point indicated by an arrow. **(C)** The sagittal T2-weighted MRI of the second case demonstrates extensive perimedullary flow voids consistent with venous congestion within the thoracic spinal cord. **(D)** The DSA findings of the second case reveal dilated tortuous perimedullary veins (the venous drainage of the fistula).

A 44-year-old man presented with a 1-year history of low back pain and leg weakness and a 1-month history of bowel and bladder dysfunction. T2-weighted magnetic resonance imaging showed large pial flow voids around the thoracolumbar spinal cord. Digital subtraction angiography (DSA) confirmed an SDAVF (Figures 1A,B).

### Case 2

A 73-year-old man presented with a 6-month history of low back pain and left leg weakness. Neurological examination revealed hyperreflexia and weakness in both legs. Large pial flow voids were apparent behind the thoracic spinal cord on T2-weighted imaging. SDAVF was confirmed on DSA (Figures 1C,D).

### Surgery

**Figure 2:**
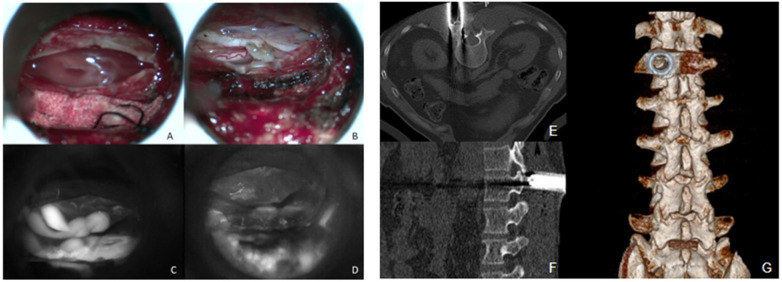
The images include intraoperative microscopic views through the microchannel and schematic diagrams of the surgical modeling. **(A,C)** The images show the arterialized veins (fistulous point and dilated draining vein) exposed after opening the dura mater (arrow), as well as the field of view under indocyanine green angiography. **(B,D)** This is the microscopic image after thermal coagulation of the fistula and its draining vein (arrow), with the appearance under indocyanine green angiography confirming absence of flow. **(E,F)** These are the CT scan images with the microchannel in place, including axial and sagittal views. **(G)** This is the three-dimensional reconstruction image obtained from intraoperative CT scanning after the placement of the surgical channel.

No special surgical contraindications were found during the examination, and the microsurgical surgery was performed. Microsurgical resection was performed in a hybrid operating room. After induction of general anesthesia, the patients were placed in the prone position and a 2 cm incision was made 2 cm to the left of midline at the L1 level. After dissection of subcutaneous tissues, the fascia and multifidus and longissimus muscles were bluntly separated to place a tubular retractor (diameter, 1.4 cm) adjacent to the L1 and L2 lamina. Intraoperative computed tomography was used to localize the level and assist with retractor placement (Figures 2E–G). Then, a high-speed burr was used to grind off the lower edge of the L1 lamina and the upper edge of the L2 lamina. The zygapophysial joints were avoided. After removing the ligamentum flavum and opening the dura, the fistula was visible (Figures 2A,C). Intraoperative indocyanine green angiography was performed to confirm the location of the fistula. After coagulating the fistula with a bipolar forceps, indocyanine green angiography was repeated to confirm closure (Figures 2B,D). Both patients' presenting symptoms—lower back pain and leg weakness—improved within 2 days after the operation. In Case 1, partial recovery of bowel and bladder function was also noted during the early postoperative period, which is an uncommon but encouraging finding given that sphincter dysfunction typically shows delayed or incomplete recovery in SDAVF patients. No complications related to the surgical procedure were observed.

## Discussion

SDAVF is the most common vascular malformation of the spinal cord, the etiology may be related to arteriovenous short circuit, spinal venous hypertension, and spinal venous blood return disorders (8) Most patients were initially misdiagnosed with other diseases leading to additional disabilities (6), so the accurate diagnosis is essential for appropriate treatment. The diagnosis first relies on MRI, which reveals a perimedullary vermiform vascular shadow, but conventional spinal angiography is still considered the gold standard for diagnosis (8, 9).Traditional spinal DSA diagnosis is an interventional procedure for patients. Therefore, it is necessary to screen patients before undergoing DSA. Rina Di Bonaventura (10) and Katarzyna Wójtowicz (11), along with their research teams, have demonstrated through studies and validation that time-resolved magnetic resonance angiography (TR-MRI) can serve as an initial screening tool for detecting spinal dural arteriovenous fistulas (SDAVF) and locating the fistulous site, with high sensitivity and specificity. For patients with a low clinical suspicion of SDAVF, TR-MRI can be used as a preliminary screening method before performing DSA for definitive diagnosis.

In addition to imaging-based auxiliary examinations, Wang et al. (12) analyzed and validated cerebrospinal fluid (CSF) and plasma samples from patients through proteomics. The study revealed that the expression levels of C4BPA and C1QA were significantly elevated in the patients' CSF. This research is the first to identify C4BPA and C1QA as potential biomarkers for spinal dural arteriovenous fistulas (SDAVF), providing new directions for the early diagnosis and pathophysiological research of SDAVF. Future studies need to validate these findings in larger cohorts of patients and explore their potential applications in disease diagnosis and treatment.

Currently, the treatment options for spinal dural arteriovenous fistulas (SDAVF) in clinical practice generally include surgical treatment and endovascular embolization. Compared to surgical treatment, endovascular embolization is a newer approach with increasing usage and success rates in clinical settings. As a more minimally invasive procedure, endovascular embolization causes less trauma to the patient, yet it still faces many challenges.Research has shown that, regardless of success rates, recurrence rates, or postoperative complication probabilities, endovascular embolization is associated with higher risks compared to surgical treatment. For example, single-center and multi-center studies by researchers such as Steinmetz et al. (1), Ma et al. (13), Singh et al. (14), Oh et al. (15), and Di Bonaventura et al. (10) highlight two important aspects: First, the importance of preoperative angiography for diagnosis is indisputable. Second, the success rate of endovascular embolization is approximately 24%–52% lower than that of surgical treatment, while the occurrence of complications is about 1.8% higher. Furthermore, extensive literature reviews by Zanin et al. (7) and Bakker et al. (16), reveal that the initial treatment failure rate of endovascular embolization is significantly higher than that of surgical treatment. Notably, the recurrence rate of embolization using Onyx can be as high as 45% (16).In summary, existing literature suggests that surgical treatment for SDAVF patients is associated with favorable success rates and lower recurrence rates in reported series, although these comparative data derive from observational studies and should be interpreted cautiously. The present two-case series is not powered to compare treatment modalities.

For the surgical treatment of spinal dural arteriovenous fistulas (SDAVFs), laminectomy is often considered essential. Sorenson et al. (17) found that SDAVFs most commonly occur in the intervertebral foramen region. Although the procedure can be performed using hemilaminectomy, Sorenson T considered factors such as dural fragility in elderly patients and the challenges posed by obesity in accessing deeper surgical fields. As a result, single-level laminectomy is the preferred surgical approach. This preference is also shared by most surgeons in current clinical practice. However, through a literature review and research analysis by Lei et al. (18), which compared unilateral laminectomy and single-level laminectomy for the treatment of spinal tumors, it was found that unilateral laminectomy significantly reduces intraoperative blood loss, shortens surgical time, and lowers the incidence of spinal deformities. In contrast, although single-level laminectomy provides a better surgical field, it can lead to spinal instability and a higher rate of postoperative complications. For us, the limitation of the surgical field in unilateral laminectomy can be addressed using our microchannel microscope expander technique. In particular, M. Afathi (19), through a study evaluating the efficacy and long-term outcomes of microchannel microsurgery for spinal canal tumors, found that this technique provides outcomes comparable to those of traditional open surgery, without significant postoperative complications. Such studies further support the effectiveness and safety of surgical treatment. Minimally invasive microscopic surgery performed using a tubular retractor not only has minimal impact on spinal bony structures but also causes less muscle damage and ligament disruption compared to open surgery, further demonstrating the safety of the procedure (20).

Currently, the application of microchannel techniques and MED (Micro Endoscopic Discectomy) technology is expanding. Notably, in addition to their widespread use in lumbar degenerative diseases, Kulkarni et al. (21) reported the use of tubular dilators in anterior cervical surgeries, including anterior cervical corpectomy and fusion (ACCF) and anterior cervical discectomy and fusion (ACDF), exploring the feasibility of applying microchannel microsurgery via the anterior cervical approach. Furthermore, Thavara et al. (22) conducted a study and retrospective analysis of the effectiveness and safety of using a tubular retractor system for intramedullary spinal cord tumors. Additionally, Fu et al. (23) demonstrated the application of microchannel microscopic surgery in cases of spontaneous spinal epidural hematoma, further proving the broad applicability of this surgical technique. To the best of our knowledge, there are very few reports describing use of a tubular retractor in SDAVF surgery.

Several technical considerations merit discussion when adapting the tubular retractor approach to SDAVF surgery. First, the most critical step in SDAVF treatment—whether open or minimally invasive—is the accurate identification of the fistulous point. In traditional open laminectomy, the wide surgical field allows comprehensive exploration of the dural surface and facilitates identification of the arterialized draining vein. The tubular retractor approach, by contrast, provides a narrower corridor that requires precise preoperative and intraoperative localization to ensure that the retractor is centered over the fistula. In our experience, this limitation is effectively mitigated by intraoperative CT guidance, which confirms the retractor position relative to bony landmarks and the target level before dural opening. This obviates the need for wider exposure while maintaining accuracy, and is particularly advantageous when the fistula has been precisely localized on preoperative DSA.

Second, the small field of view inherent to the tubular approach presents specific challenges. In our second case, the thoracic location and the presence of extensive perimedullary flow voids required careful orientation under the microscope to distinguish the arterialized draining vein from normal vasculature. The use of intraoperative ICG angiography was indispensable in this regard, providing real-time confirmation of the fistula location before coagulation and verifying complete obliteration afterward. The visualization of the temporal sequence of venous filling—from early abnormal opacification to normalized flow after coagulation—serves as a functional endpoint that compensates for the limited anatomical exposure.

Third, the choice between a standard open approach and a tubular retractor approach involves a trade-off between surgical exposure and tissue preservation. Traditional single-level laminectomy, as advocated by Sorenson et al. (17), provides excellent visualization and is preferred by many surgeons, particularly in elderly patients with dural fragility or in obese patients where the surgical field depth poses a challenge. However, the associated disruption of the posterior tension band—paraspinal muscles, ligaments, and bony structures—may contribute to postoperative pain, delayed mobilization, and potential long-term spinal instability. The tubular retractor approach, by bluntly splitting rather than transecting the paraspinal musculature and by requiring only a limited laminotomy rather than a full laminectomy, substantially reduces this collateral tissue injury. This consideration is particularly relevant for SDAVF patients who often have preexisting degenerative spinal changes and may be at increased risk for post-laminectomy deformity.

With regard to the literature on tubular access specifically for SDAVF, reports remain sparse. To date, the tubular retractor technique has been more extensively described for degenerative lumbar conditions, intradural spinal tumors (19, 22), and spinal epidural hematoma evacuation (23). Its application to spinal vascular lesions, including SDAVF, represents a logical extension of the technique, predicated on the same principles of minimizing approach-related morbidity while achieving the primary surgical objective—in this case, fistula obliteration. The favorable outcomes in our two cases, consistent with those reported for tubular approaches in other spinal pathologies, support the feasibility and safety of this technique for carefully selected SDAVF patients.

Finally, the adjunctive roles of intraoperative CT and ICG angiography deserve emphasis. Intraoperative CT provides precise verification of the retractor position relative to the target level, compensating for the limited visual field and reducing the risk of wrong-level surgery—a recognized concern in minimally invasive spine procedures. ICG angiography, in turn, provides dynamic, real-time assessment of vascular anatomy and flow patterns that is not obtainable with static CT imaging alone. The integration of these two modalities creates a workflow in which CT ensures accurate anatomical targeting and ICG ensures accurate physiological targeting—an integrated navigation-and-confirmation paradigm that enhances the safety profile of the minimally invasive approach.

## Conclusion

Microsurgery for SDAVF obliteration can be performed via a tubular retractor approach. This technique reduces surgical injury to paraspinal structures and shortens hospital stay. Larger outcome studies with long-term follow-up are warranted.

## Data Availability

The original contributions presented in the study are included in the article/Supplementary Material, further inquiries can be directed to the corresponding authors.
